# A Comprehensive Review on Medium- and Long-Chain Fatty Acid-Derived Metabolites: From Energy Sources to Metabolic Signals

**DOI:** 10.3390/metabo16010045

**Published:** 2026-01-04

**Authors:** Jin-Byung Park, Sungyun Cho, Sung-Joon Lee

**Affiliations:** 1 Department of Food Science and Biotechnology, Ewha Womans University, Seoul 03760, Republic of Korea; jbpark06@ewha.ac.kr; 2 Department of Pharmacology, Korea University College of Medicine, Seoul 02841, Republic of Korea; sungyuncho@korea.ac.kr; 3 Department of Food Bioscience & Technology, College of Life Sciences & Biotechnology, Korea University, Seoul 02841, Republic of Korea

**Keywords:** medium-chain fatty acids, long-chain fatty acids, fatty acid derivatives, biotransformation, gut microbiome, ectopic olfactory receptors (ORs), ectopic bitter taste receptors (TAS2Rs)

## Abstract

Medium- and long-chain fatty acids (MLFAs) are increasingly recognized not only as metabolic substrates but also as precursors of diverse bioactive metabolites generated through host and microbial transformations. Recent advances in analytical chemistry and microbiome research have revealed that gut microorganisms catalyze extensive modifications of dietary MLFAs—producing hydroxylated, conjugated, and keto-fatty acids with enhanced potency toward host receptors. These metabolites exhibit dual activity on classical metabolic receptors, including FFAR1/4 and PPARα/γ, as well as ectopically expressed chemosensory receptors such as olfactory receptors (ORs) and bitter taste receptors (TAS2Rs). This expanded receptor landscape establishes a previously unrecognized chemosensory–metabolic axis that integrates dietary signals, microbial metabolism, and host physiology. Microbial MLFA derivatives such as 10-hydroxyoctadecenoic acid and conjugated linoleic acid regulate incretin secretion, adipogenesis, macrophage polarization, and intestinal barrier function through coordinated activation of FFARs and PPARs. Concurrently, dicarboxylic acids such as azelaic acid activate Olfr544 to modulate lipolysis, ketogenesis, GLP-1 release, and feeding behavior. TAS2Rs also sense oxidized lipids, linking lipid metabolism to immune regulation and enteroendocrine signaling. Collectively, these pathways highlight the microbiome as a metabolic transducer that converts dietary lipids into signaling molecules influencing endocrine, immune, and gut–brain circuits. Understanding the mechanisms governing MLFA bioconversion and receptor engagement provides new opportunities for therapeutic and nutritional intervention. Targeting ORs and TAS2Rs, engineering probiotics to enhance beneficial FA-derived metabolites, and developing receptor-selective synthetic analogs represent promising strategies. Future progress will require integrative approaches combining physiology, biochemistry, metabolomics, and microbial genomics to elucidate receptor specificity and host variability.

## 1. Introduction

Medium- and long-chain fatty acids (MLFAs), fundamental components of dietary fats and oils, serve essential roles not only as energy substrates and structural elements of cell membranes. However, accumulating evidence over the past two decades has established that these fatty acids also function as potent signaling molecules [1] Classical MLFA signaling is mediated by two major receptor families: G protein-coupled receptors such as free fatty acid receptor (FFAR)1/4 (GPR40/120) [2], and the peroxisome proliferator-activated receptors (PPARs α, γ, δ) [3] At the cell surface, FFAR1/4 respond to extracellular fatty acids (FAs) to regulate incretin hormone secretion (GLP-1, GIP), insulin sensitivity, and inflammatory pathways [2] Intracellularly, MLFA ligands engage PPARs, the active forms are transcription factors, to control gene expression programs that govern lipid oxidation, adipogenesis, and inflammatory signaling [3]

The clinical importance of these signaling pathways is exemplified by PPAR agonists. Thiazolidinediones (TZDs), which act on PPARγ, have proven effective in improving insulin sensitivity in type 2 diabetes, whereas fibrates target PPARα to reduce triglycerides and modulate lipid profiles. Nevertheless, their clinical use has been tempered by adverse effects related to receptor subtype promiscuity, off-target activation, species differences, and tissue-specific heterogeneity in receptor expression [4,5,6,7]. These limitations have motivated intensive efforts to identify novel fatty-acid-responsive targets with improved selectivity and fewer pleiotropic effects.

A rapidly expanding area of research concerns the metabolic transformation of MLFAs within the gastrointestinal tract. During digestion, dietary MLFAs undergo complex enzymatic and microbial bioconversion, giving rise to a diverse portfolio of medium- and long-chain fatty acid (LMFA) metabolites, including oxygenated lipids, hydroxy-FAs, short-chain derivatives, and branched-chain species [8,9,10]. Recent advances in lipidomics and microbiome metabolite profiling have revealed that these microbial transformations expand the chemical diversity of host-accessible fatty acids far beyond the dietary inputs. Notably, several microbially derived LMFA metabolites exhibit biological potencies equal to or greater than their parent lipids, influencing immune tone, epithelial homeostasis, lipid metabolism, and gut–brain communication [8,9,11,12,13].

Parallel to these discoveries, an unexpected class of FA-responsive receptors has gained attention: ectopically expressed chemosensory receptors, including olfactory receptors (ORs) and bitter taste receptors (TAS2Rs). Once thought to function exclusively in the nose and tongue, respectively, these receptors are now known to be widely expressed in metabolic, immune, gastrointestinal, and endocrine tissues [14]. Many ORs and TAS2Rs respond to medium- and long-chain FAs or their metabolites, suggesting an additional chemosensory layer through which the host monitors lipid-derived signals. Activation of these receptors has been linked to regulation of mitochondrial function, adipocyte thermogenesis, hormone secretion, smooth muscle contraction, epithelial barrier function, and immune responses. This expanding paradigm suggests that dietary FAs, together with their microbiota-derived metabolites, engage a distributed network of classical and non-classical receptors to fine-tune metabolic and physiological outcomes in a tissue-specific manner.

In this review, we summarize current knowledge on MLFA bioconversion by gut microorganisms, characterize the structural and functional diversity of resulting metabolites, and highlight the emerging roles of ectopic chemosensory receptors in mediating their bioactivity. We further discuss the growing impact of computational tools, including lipid-ligand docking, OR/TAS2R structure prediction, and metabolic pathway inference, which are accelerating the discovery of novel FA-derived signaling molecules and their physiological targets.

## 2. LMFAs in Gut: Bioconversion of Dietary Fats and Prediction of Metabolism in Gut

### 2.1. Digestion and Biotransformation of Dietary Fats

Dietary fats undergo a sequence of physicochemical and enzymatic transformations before reaching the colon. In the oral cavity, mastication disperses lipids while salivary phospholipids initiate partial emulsification. Lingual lipases hydrolyze triglycerides to free fatty acids, mainly MLFAs. Long-chain fatty acids (LCFAs, C7-C21) activate FFAR4 [15] and LCFA derivatives such as α-linoleic acid have a bitter taste that interacts with TAS2Rs expressed in tongue and oral tissues [16]. In the stomach, acidification and gastric lipases generate additional MLFAs and sn-1,2-diacylglycerols [17].

**Figure 1:**
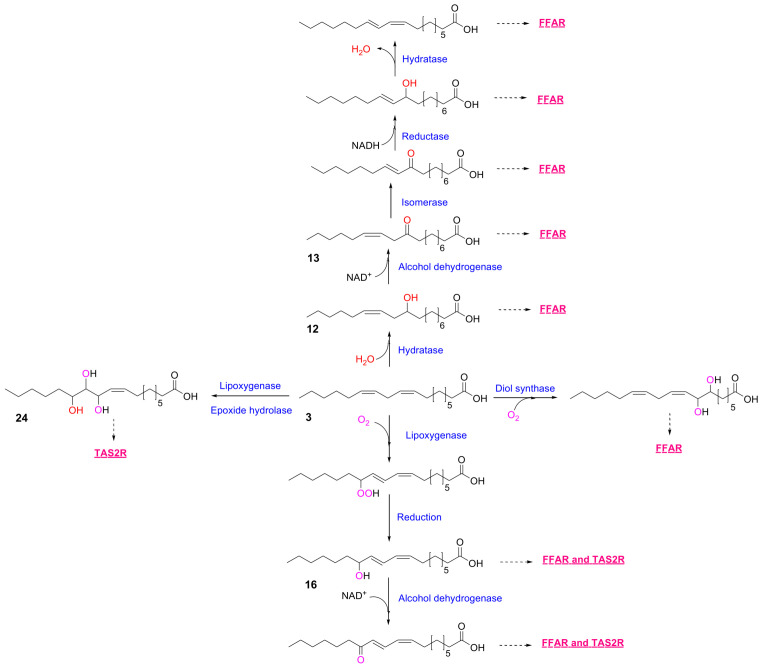
***Biotransformation pathways of linoleic acid.*** Linoleic acid can be converted into CLA via 10-hydroxyoctadec-12*Z*-enoic acid (**12**) and 10-keto-octadec-12*Z*-enoic acid (**13**) [10]. Linoleic acid can be also transformed into 13-hydroxyoctadeca-9*Z*,11*E*-dienoic acid (**16**) [38,47]. See Table 1 for EC_50_ values of the fatty acids. The blue words indicate the enzymes, which catalyze the each reaction steps. The red words indicate the taste receptors, which could be activated by the each metabolites. FFAR: free fatty acid receptor, TAS2R: bitter taste receptor.

Upon entering the duodenum, bile acids and phospholipids efficiently emulsify lipids, providing a substrate interface for pancreatic lipase–colipase complexes. Hydrolysis produces MLFAs and 2-monoacylglycerols, which are absorbed primarily in the proximal small intestine. These intermediates stimulate enteroendocrine cells to release cholecystokinin (CCK), partly through activation of FFAR1/4 in the small intestine [18,19]. A fraction of undigested or partially digested MLFAs escapes absorption and reaches the colon. Here, microbial communities carry out extensive biotransformations, producing hydroxylated, conjugated, and keto-derivatives that profoundly influence host metabolism (Figure 1).

The human gut microbiome greatly expands the repertoire of metabolic reactions beyond those encoded by the host genome, thereby shaping host physiology in profound ways. The chemical diversity generated by microbial metabolism of dietary lipids produces signaling molecules that modulate host energy homeostasis, immune function, and metabolic health [20,21,22,23]. Recent advances in bioinformatics and genome-scale metabolic modeling have enabled the systematic prediction of gut microbial metabolites and the reconstruction of their metabolic pathways, providing a framework to link diet–microbe–host interactions at a systems level [10].

**Figure 2:**
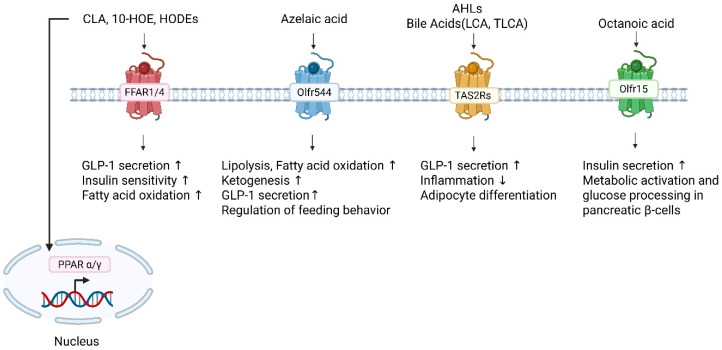
***Ligand–receptor interactions linking lipid-derived metabolites, olfactory and taste receptors, and metabolic signaling pathways.*** CLA, 10-HOE, and HODEs activate FFAR1/4 to enhance GLP-1 secretion, insulin sensitivity, and fatty-acid oxidation. These fatty acid derivatives bind and activate PPAR α/γ signaling to regulate metabolism and inflammation. Azelaic acid, 9-carbon dicarboxylic acid, activates an olfactory receptor Olfr544, promoting lipolysis, fatty-acid oxidation, ketogenesis, and GLP-1 secretion, and regulates feeding behavior. AHLs and bile acids (LCA, TLCA) stimulate TAS2Rs, resulting in increased GLP-1 secretion, reduced inflammation, and modulation of adipocyte differentiation. The TAS2R isoforms activated by these bitter tastants are not yet clarified. Octanoic acid activates Olfr15, leading to increased insulin secretion and metabolic activation in pancreatic β-cells. Collectively, these receptor–ligand pathways integrate microbial and lipid metabolites with host metabolic regulation via PPAR-dependent transcriptional responses. FFAR1/4 (red), Olfr544 (blue), TAS2Rs (yellow), Olfr15 (green), and PPARα/γ (pink) are shown in different colors to indicate receptor classes.

Among the best-characterized microbial lipid transformations is the conversion of linoleic acid (LA; C18:2, n-6) into conjugated linoleic acid (CLA) (Figure 1) [10], a known agonist of peroxisome proliferator-activated receptor alpha (PPARα) (Figure 2). Lactic acid bacteria (e.g., *Lactobacillus plantarum*, *Lactobacillus reuteri*) catalyze this transformation via fatty acid double bond hydratases, alcohol dehydrogenases, and isomerases [10,24,25,26,27,28,29,30,31,32] Key intermediates include 10-hydroxyoctadec-12*Z*-enoic acid (10-HOE) (**12**) and 10-keto-octadec-12*Z*-enoic acid (10-KOE) (**13**) (Table 1 and Figure 1). Of note, increased levels of 10-HOE were found in the ileum and cecum, where *Lactobacillus* is preferentially resides [13], suggesting its in vivo microbial origin. Functionally, 10-HOE activates both FFAR1 [12,33,34] and FFAR4 (Figure 2 and Table 1) [13,35], in addition to PPARγ, implicating it as a multifunctional signaling lipid at the host–microbe interface. The detection of 10-HOE suggests the existence of parallel bioconversions from other dietary polyunsaturated fatty acids [36,37,38] For example, oleic acid (C18:1) is converted to 10-hydroxyoctadecanoic acid, α-linolenic acid (C18:3, n-3) to 10-hydroxyoctadec-12,15-dienoic acid [39,40,41], and γ-linolenic acid (C18:3, n-6) to 10-hydroxyoctadec-6,12-dienoic acid [42,43], respectively. Such transformations highlight the substrate promiscuity of microbial hydratases and the metabolic diversity of gut-derived hydroxylated fatty acids.

In addition to hydratase-driven pathways, microbial lipoxygenases (LOXs) produce hydroxy and keto fatty acids thus mediate the oxidation of linoleic acid into hydroxyoctadecadienoic acids (HODEs) (Figure 1) [38,44]. With reducing agents such as cysteine, linoleic acid (**3**) can be converted into 9-hydroxyoctadeca-10*E*,12*Z*-dienoic acid (**10**) and 13-hydroxyoctadeca-9*Z*,11*E*-dienoic acid (**16**). These metabolites exhibit potential agonist activity for FFAR1, FFAR4 (Figure 2 and Table 1), and selected TAS2Rs [16,35], thereby broadening the range of receptors responsive to microbial lipids. Furthermore, hydroxy fatty acids undergo secondary oxidation by microbial alcohol dehydrogenases to yield keto-fatty acids (e.g., oxidation of 10-HOE (**12**) into 10-KOE (**13**)). Keto-derivatives of MLFA are recognized ligands for PPARγ [24,45,46], linking microbial lipid oxidation to nuclear receptor signaling that governs adipogenesis and glucose metabolism.

Another interesting point would be the relationship between fatty acid structure and its agonist activity. The number and position of hydroxyl groups as well as the degree of unsaturation of fatty acids played a key role in their agonist activities. For instance, 8,11-dihydroxyoctadec-9*Z*-enoic acid exhibited significantly greater Ca^2+^ response in the FFAR1/4-expressing cells as compared to the endogenous agonists (e.g., linoleic acid and docosahexaenoic acid) and the mono-hydroxy fatty acids (e.g., 10-HOE), forming hydrogen bond interactions with residues in the ligand-binding pockets of receptors.

### 2.2. In Silico Predictions of MLFA Metabolites-Receptor Interactions

MLFA metabolites in gut may be produced chemically; however, themajority of them are formed by biotransformation of gut microbiome particularly in the colon. The integration of in silico metabolic network in systems biology with receptor–ligand modeling reconstructs a powerful approach to predict host responses to MLFA metabolites. Genome-scale metabolic models allow simulation of polyunsaturated fatty acid metabolism by specific taxa (e.g., *Lactobacillus*, *Bifidobacterium*), predicting production fluxes of CLA, HODEs, and keto-fatty acids under different dietary contexts. Coupled with computational docking, these predictions can identify which microbial metabolites are most likely to activate host receptors, thereby linking microbial ecology to host signaling. For instance, 10-HOE (**12**), which is produced from linoleic acid by lactic acid bacteria [32] was shown to activate FFAR1 and FFAR4 as a dual agonist (Figure 2) [13,34,35] while 13-hydroxyoctadeca-9*Z*,11*E*-dienoic acid (**16**) preferentially activated FFAR4 (Table 1) [35]. In addition, 13-hydroxyoctadeca-9*Z*,11*E*-dienoic acid (**16**) may activate TAS2Rs, since 13-hydroxyoctadeca-9*Z*,11*E*-dienoic acid (**16**) is a major bitter compound in pea-protein isolates [16] Thus, it will be of great interest to examine the cellular concentrations, binding affinity, and physiological functions identifying TAS2R isoforms activated by this compound in the future. Such predictions can guide targeted dietary interventions or probiotic supplementation strategies as well.

Microbial FA derivatives exert diverse effects on host physiology: CLA and microbially derived hydroxy fatty acids act as ligands for PPARα and PPARγ (Figure 2), thereby enhancing mitochondrial FA oxidation and contributing to improved lipid metabolism and insulin sensitivity [48,49,50,51]. Activation of the free fatty acid receptors FFAR1/4 promotes secretion of incretin hormones, including GLP-1 and GIP [52,53], as well as satiety-associated peptides such as CCK [54,55] and PYY [56,57], linking microbial lipid metabolites to enteroendocrine signaling. In the immune compartment, hydroxy fatty acids modulate macrophage polarization and reinforce intestinal barrier integrity through PPARγ-dependent pathways [58,59,60,61]. Furthermore, engagement of FFAR1/4 by microbial lipid derivatives may influence gut–brain communication, particularly neural circuits regulating appetite and reward processing [62,63].

The protein structures of olfactory and bitter taste receptors have only recently begun to be elucidated. High-resolution structures are now available for OR51E2 and TAS2R14. Prior to these reports, in silico modeling of chemosensory GPCRs depended largely on rhodopsin-based homology templates, and the resulting structural predictions required extensive validation through site-directed mutagenesis. With the emergence of experimentally resolved OR and TAS2R structures, however, olfactory receptor modeling can now utilize OR51E2 as a receptor-specific and physiologically relevant template. The availability of native OR structures greatly improves the accuracy and reliability of structural predictions, ligand-docking simulations, and receptor–ligand interaction analyses, representing a significant advance over earlier rhodopsin-based models.

### 2.3. Chemosensory Receptors as Novel Fatty Acid Targets: Expanding the Landscape of Lipid Signaling

#### 2.3.1. Olfactory Receptors as Metabolic Sensors of Fatty Acids

Several ectopically expressed ORs have recently been identified as functional sensors for MLFA metabolites. Medium-chain fatty acids are detected by Olfr15, which is highly expressed in pancreatic β-cells [64]. Acute Olfr15 activation by octanoic acid potentiates glucose-stimulated insulin secretion (GSIS) through PLC–IP_3_–Ca^2+^ signaling (Figure 2), whereas chronic stimulation enhances glucokinase expression via an IP_3_–CaMKK/CaMKIV pathway [65]. Olfr15 is also expressed in adipocyte and regulates adipogenesis. Notably, reduced Olfr15 expression in diabetic mice underscores its potential relevance in metabolic disease [64].

Importantly, the gut microbiome expands the ligand pool for ORs by converting dietary FAs into hydroxylated and conjugated derivatives. Azelaic acid (AzA), a nine-carbon dicarboxylic acid is a potential microbial metabolite mainly formed from unsaturated fatty acids such as oleic and linoleic acid [66]. Several AzA producing pathways are known. First is oxidative cleavage of the double bond in oleic acid together with the formation of shorter aldehydes and ketones [66]. Lipoxygenase-like microbial enzymes and host reactive oxygen species (ROS)-driven lipid peroxidation can also participate in this pathway. Second, AzA can be produced from unsaturated fatty acids by lipoxygenase or Baeyer–Villiger monooxygenase as a key enzyme [38,67]. Alternatively, AzA is also produced by both host enzymes (cytochrome P450–like ω-hydroxylases) and microbial oxidoreductases contribute to the conversion of medium-chain fatty acids [68]. The hydroxylation of ω-carbon of medium chain fatty acids, which are then further oxidized via alcohol and aldehyde dehydrogenases into dicarboxylic acids, produce AzA [69,70].

Though its microbial producers remain unconfirmed, candidate taxa such as *Clostridium* and *Bacteroides* may also indirectly contribute to its biosynthesis [71]. Several gut- microbes (e.g., *Pseudomonas*, *Candida*, *Malassezia*) also harbor ω-oxidation and fatty acid–cleaving enzymes that facilitate the production of AzA from unsaturated LCFAs [68,72,73,74]. These microbial transformations complement host peroxisomal metabolism, expanding the pool of gut-derived dicarboxylic acids.

Azelaic acid has been implicated in activating AMP-activated protein kinase (AMPK) [75], modulating PPARγ [76,77], and improving mitochondrial function [78]. Within the gut–liver axis, it may influence lipid homeostasis, oxidative stress responses, and inflammatory pathways. Moreover, azelaic acid itself may act as a microbial growth modulator, shaping the composition of the gut microbiome. While minor in concentration in gut, AzA has reported roles in modulating inflammation and cellular proliferation, warranting further exploration of its gut microbial origins and systemic functions.

Interestingly, an olfactory receptor 544 (Olfr544) recognizes AzA as an endogenous ligand and accumulating evidence indicates that Olfr544 activation orchestrates endocrine, metabolic, and immune functions across multiple organs. Olfr544 is highly expressed in liver, adipose tissue, skeletal muscle, pancreas and gut, where its activation by AzA mediates profound metabolic effects [79,80,81]. Thus, oral administration of AzA in mice reduces adiposity shifting fuel preference to fat (Figure 2) [79]. These effects are negated in Olfr544-deficient mice demonstrating AzA effect is Olfr544-dependent.

Within the brain, Olfr544 is expressed in cortical and hippocampal neurons, with additional localization in glial and endothelial cells. Its expression profile changes with age and disease, with altered patterns observed in transgenic Alzheimer’s disease-like mice, particularly in proximity to amyloid plaques [82]. Functionally, Olfr544 also regulates feeding behavior. AzA treatment increased CCK secretion and reduced preference for high-fat diet (HFD) in mice. These effects were abolished in Olfr544 knockout animals, and pharmacological blockade of CCK receptors negated AzA-induced reductions in fat preference, implicating an Olfr544–CCK axis in the regulation of dietary choices (Figure 2) [81]. Collectively, these findings establish Olfr544 as a versatile chemosensory receptor that integrates AzA signals with systemic metabolic regulation. The abrogation of these effects in Olfr544-deficient mice underscores its central role as a molecular hub in coordinating metabolic responses across multiple organs.

#### 2.3.2. Bitter Taste Receptors as Peripheral Sentinels for Fatty Acid Metabolites

Complementing the OR system, TAS2Rs act as peripheral sentinels for hydroxylated, epoxidized, and oxidized FA derivatives in extra-oral tissues [83]. For instance, bile acids and cholesterol emerge as key players. Specifically, bile acids such as lithocholic acid and taurolithocholic acid robustly activate TAS2Rs, modulating digestion, immune responses, and inflammation via GLP-1 secretion (Figure 2) [84,85]. Cholesterol enhances TAS2R14 activity by occupying its orthosteric site, with concentration-dependent activation, stabilizing receptor structure and aiding airway signaling [86]. Additionally, lipid-derived ligand like progesterone [87] expands this repertoire, potentially regulating metabolic and developmental processes as endogenous modulators [88] further expand the ligand repertoire, suggesting a broad interaction between lipid metabolites and TAS2Rs in extra-oral tissues, potentially influencing metabolic regulation [89].

Microbiota-derived metabolites such as acyl-homoserine lactones (AHLs) can act as TAS2R ligands, demonstrating that microbial activity directly influences chemosensory receptor activation in the gut [90]. As quorum-sensing molecules produced by intestinal microbes, AHLs not only mediate bacterial communication but also modulate epithelial barrier function, inflammatory pathways, and host immune responses [83,91,92]. TAS2Rs have been reported to be expressed in adipocytes, where they contribute to the regulation of adipocyte differentiation [93].

Given that certain fatty acids function as TAS2R ligands [94], it is plausible that adipocyte-derived fatty acid metabolites modulate lipolysis and adipogenesis via TAS2R-mediated signaling pathways. Genetic variations in TAS2Rs influence not only receptor sensitivity but also dietary fat preference and metabolic outcomes. Graham et al. (2021) [95] showed that there is an inverse correlation between bitter taste sensitivity and saturated fat intake as individuals carrying both a “non-taster” TAS2R38 haplotype (AVI) consumed significantly more saturated fat examine among 88 Caucasian participants, illustrating how chemosensory genotypes intersect with nutritional behavior.

## 3. Perspectives and Therapeutic Potential

### 3.1. Chemosensory-Metabolic Integration of Fatty Acid Metabolites

Recognition that FA metabolites activate ectopically expressed ORs and TAS2Rs has revealed a previously unappreciated chemosensory dimension of metabolic regulation. This integrated chemosensory–metabolic network enables the host to sense not only primary dietary FAs but also their downstream metabolites, many of which arise from microbial biotransformation or oxidative stress. The hormonal activity of these FAs and their derivatives emerge as central messengers in a dynamic surveillance system linking diet, microbiota, and host physiology. A key conceptual insight is that FA derivatives frequently act as dual ligands for both chemosensory receptors (e.g., ORs, TAS2Rs) and classical metabolic receptors such as PPARs and FFAR1/4. This convergence enables coordinated endocrine, metabolic, and immune responses. For example, hydroxylated linoleic acid derivatives can simultaneously activate FFAR4 to stimulate GLP-1 release and PPARγ to regulate adipogenesis, demonstrating how receptor cross-receptor integration aligns energy balance with immune function.

### 3.2. Microbiome as a Metabolic Signaling Hub

A second major conceptual advance is the recognition of the gut microbiome as a biochemical transducer. Gut microbes convert dietary polyunsaturated fatty acids into hydroxylated, conjugated, and keto-derivatives with potent bioactivities. These microbial metabolites engage Ors, TAS2Rs FFARs, and PPARs across multiple tissues-including the gut, liver, adipose tissue, immune cells, and the nervous system-affecting enteroendocrine hormone secretion to macrophage polarization. At broader biological scale, this chemosensory–metabolic axis regulates cellular responses such as Olfr15-mediated insulin secretion in pancreatic β-cells and TAS2R-dependent incretin release in L cells [96,97] At the organismal level, receptor polymorphisms such as TAS2R38 and CD38 interact with dietary input to shape fat preference and nutrient intake, while systemic receptor cross-activation orchestrates endocrine, metabolic, and gut–brain circuits governing appetite and reward.

### 3.3. Translational Opportunities Methodological Challenges

Understanding the chemosensory dimension of FA signaling offers promising translational opportunities. Targeting ORs and TAS2Rs may offer strategies to modulate hormone release, appetite control, immune responses, or inflammation. Microbiome-based approaches-such as prebiotics or probiotics engineered to enhance beneficial FA metabolite production-and synthetic lipid analogs with refined receptor specificity represent additional therapeutic routes. However, significant challenges remain. Ligand promiscuity and receptor specificity, and broad tuning of many chemosensory receptors complicate mechanistic interpretation. Advances in structural biology, in silico docking, mutagenesis, and high-throughput ligand screening are needed to resolve receptor–ligand interactions at atomic resolution. Comprehensive metabolomics integrated with strain-level microbial genomics will be essential to identify microbial producers of key FA-derived metabolites, while genetic variability in FFARs, PPARs, ORs, and TAS2Rs underscores the importance of personalized approaches in nutrition and metabolic medicine.

A key message regarding the clinical relevance of this emerging field is that ectopic olfactory and bitter taste receptors should be considered as potential target proteins when investigating the mechanisms of action of MLFA-derived metabolites. In addition, the relative binding affinity and functional activity of these metabolites toward ectopic chemosensory receptors need to be systematically compared with their activity at classical fatty acid receptors such as FFAR1/4 and PPARs. It is entirely plausible that certain novel MLFA-derived metabolites may selectively activate ectopic olfactory or bitter taste receptors rather than FFAR1/4 or PPARs. If this is the case, the molecular novelty and physiological significance of these metabolites would become far more apparent.

Importantly, when studying the mechanisms and bioactivities of MLFA-derived compounds, researchers should evaluate not only their potential to act as PPAR or FFAR1/4 agonists but also the possibility that ectopic olfactory receptors or TAS2Rs may serve as their primary molecular targets. Identifying the receptor with the highest binding affinity and functional responsiveness will be essential for elucidating tissue-specific actions, predicting efficacy, and anticipating potential off-target effects or adverse outcomes. Such considerations will be critical in guiding the design and interpretation of future preclinical studies and should be carefully integrated into strategies aimed at eventual human application. Future research will ultimately determine the clinical implications and therapeutic potential of MLFA-derived metabolites.

## 4. Conclusions

In summary, the convergence of classical FA signaling pathways with ectopic chemosensory receptor biology establishes a new paradigm in host–microbiome communication. By integrating metabolic, immune, endocrine, and neural responses, this expanded framework highlights the therapeutic potential of harnessing FA–receptor interactions to prevent or treat metabolic and inflammatory diseases.

## Figures and Tables

**Table 1:** EC_50_ values of FA ligands for GPR40 and GPR120 [31,35].

No.	Fatty Acid Ligand	EC_50_ (µM)	
		GPR40	GPR120
**1**	stearic acid (18:0)	ND	ND
**2**	oleic acid (18:1, n-9)	27.5	17.3
**3**	linoleic acid (18:2, n-6)	12.1	7.02
**4**	γ-linolenic acid (18:3, n-6)	3.18	2.01
**5**	α-linolenic acid (18:3, n-3)	2.27	2.04
**6**	arachidonic acid (20:4, n-6)	9.54	8.20
**7**	eicosapentaenoic acid (20:5, n-3)	5.44	2.73
**8**	docosahexaenoic acid (22:6, n-3)	2.42	0.91
**9**	8-hydroxyoctadec-9*Z*-enoic acid	12.7	7.66
**10**	9-hydroxyoctadeca-10*E*,12*Z*-dienoic acid	3.04	3.04
**11**	10-hydroxyoctadecanoic acid	ND	ND
**12**	10-hydroxyoctadec-12*Z*-enoic acid	2.18	2.30
**13**	10-keto-octadec-12*Z*-enoic acid	3.61	3.40
**14**	12-hydroxyoctadec-9*Z*-enoic acid	11.7	3.58
**15**	13-hydroxyoctadec-9*Z*-enoic acid	9.83	2.04
**16**	13-hydroxyoctadeca-9*Z*,11*E*-dienoic acid	6.90	1.07
**17**	5,8-dihydroxyoctadec-9*Z*-enoic acid	9.77	2.10
**18**	6,8-dihydroxyoctadec-9*Z*-enoic acid	1.50	2.51
**19**	7,8-dihydroxyoctadec-9*Z*-enoic acid	1.28	2.69
**20**	8,11-dihydroxyoctadec-9*Z*-enoic acid	0.30	0.64
**21**	8,12-dihydroxyoctadec-9*Z*-enoic acid	4.78	0.43
**22**	10,12-dihydroxyoctadecanoic acid	ND	ND
**23**	10,13-dihydroxyoctadecanoic acid	ND	ND
**24**	11,12,13-trihydroxyoctadec-9*Z*-enoic acid	ND	ND

ND, not detected (signal below the limit of detection).

## Data Availability

No new data were created or analyzed in this study.
